# Supplementary material for: Drug-Resistant Epimutants Exhibit Organ-Specific Stability and Induction during Murine Infections Caused by the Human Fungal Pathogen Mucor circinelloides
Source: mBio. 2019 Nov 5;10(6):e02579-19. doi: 10.1128/mBio.02579-19 (PMC6831780; doi:10.1128/mBio.02579-19)
Supplement: TABLE S1 [file mBio.02579-19-st001.pdf]

Strain	Organ	Total colonies patched	FK506-resistant colonies	Ratio of resistance
SCV522 (*fkbA* epimutant)	Brain	57	27	47.4%
	Liver	150	148	98.7%
	Spleen	132	131	99.2%
	Kidney	148	145	98.0%
	Lung	1	1	100.0%
1006PhL (WT)	Brain	88	0	0.0%
	Liver	150	0	0.0%
	Spleen	150	0	0.0%
	Kidney	156	0	0.0%
	Lung	4	0	0.0%
1006PhL spores (without *in vivo* passage)	N/A	150	0	0.0%
SCV522 spores (without *in vivo* passage)	N/A	150	150	100.0%

Table S1: Colony counts for ratios plotted in figure 2B.
