# Supplementary figures and images for: Drug-Resistant Epimutants Exhibit Organ-Specific Stability and Induction during Murine Infections Caused by the Human Fungal Pathogen Mucor circinelloides
Source: mBio. 2019 Nov 5;10(6):e02579-19. doi: 10.1128/mBio.02579-19 (PMC6831780; doi:10.1128/mBio.02579-19)

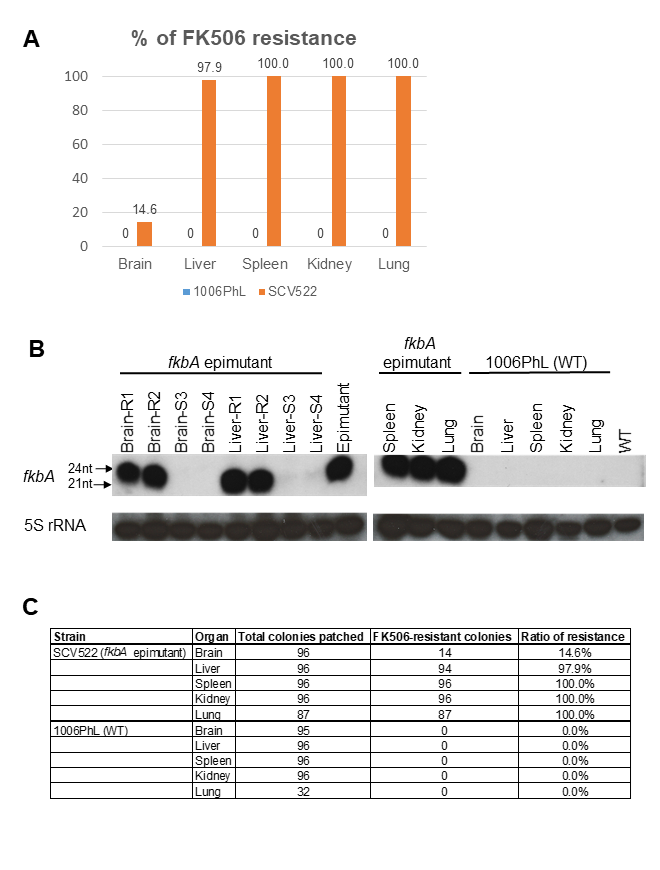

Supplement: FIG S1 [file mBio.02579-19-sf001.tif]

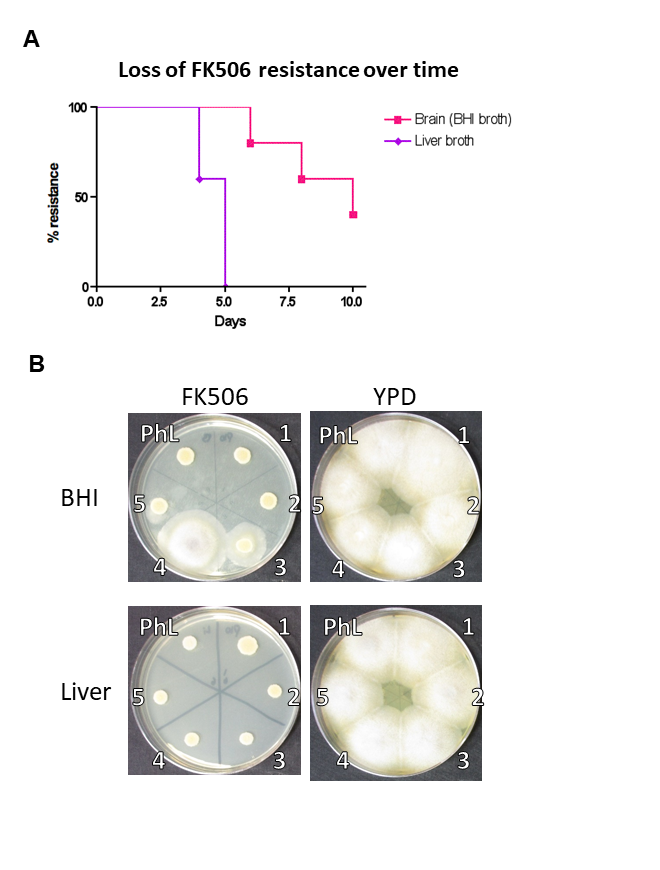

Supplement: FIG S2 [file mBio.02579-19-sf002.tif]
