# Supplementary material for: Drug-Resistant Epimutants Exhibit Organ-Specific Stability and Induction during Murine Infections Caused by the Human Fungal Pathogen Mucor circinelloides
Source: mBio. 2019 Nov 5;10(6):e02579-19. doi: 10.1128/mBio.02579-19 (PMC6831780; doi:10.1128/mBio.02579-19)
Supplement: TABLE S2 [file mBio.02579-19-st002.pdf]

Table S2. Primers used in this study

Name	Sequence	Use
JOHE40635	ACCCTGCTACAGTCGATGCT	*M. circinelloides* f. *circinelloides* 1006PhL *fkbA* sRNA probe synthesis
JOHE40638	TAATACGACTCACTATAGGGCTGTACCATATGGACATTGTG	*M. circinelloides* f. *circinelloides* 1006PhL *fkbA* sRNA probe synthesis (w/ T7 promoter)
JOHE37682	TAATACGACTCACTATAGGGAGCTACGGCCATACAATGTTG	5S rRNA probe synthesis (T7 promotor included)
JOHE37683	TAATACGACTCACTATAGGGGAACTACAGCAACCAGTATTCCCA	5S rRNA probe synthesis (T7 promotor included)
JOHE38278	GAGGAATGAGACCGGGGTAACCAC	24nt for size standard on sRNA blots
JOHE42163	AGATCCACGATCACGAGATGA	21nt for size standard on sRNA blots
